# Co-occurrence of *fosA5*, *bla*_SHV-145_ and *bla*_OXA-48_ among a *Klebsiella pneumoniae* high-risk ST16 from a tertiary hospital in China: focusing on the phylogeny of OXA-48 genes from global *Klebsiella pneumoniae* isolates

**DOI:** 10.1007/s42770-021-00572-6

**Published:** 2021-08-17

**Authors:** Yanmei Sun, Wei Chen, Shiwei Wang, Xiaoli Cao

**Affiliations:** 1 Key Laboratory of Resources Biology and Biotechnology in Western China, Ministry of Education, College of Life Science, Northwest University, Xi’an, 710069 People’s Republic of China; 2 Clinical Research Center, the Second Hospital of Nanjing, Nanjing University of Chinese Medicine, Nanjing, 210003 People’s Republic of China; 3 Department of Laboratory Medicine, Nanjing Drum Tower Hospital, the Affiliated Hospital of Nanjing University Medical School, Zhongshan Road, 321#, Gulou District, Nanjing, Jiangsu People’s Republic of China

Since the OXA-48-type carbapenem-hydrolyzing class D β-lactamase was reported in a *Klebsiella pneumoniae* isolate from Istanbul (Turkey) in 2001 [1], this carbapenemase has been widely distributed among *Enterobacterales*, with significant geographical differences [2, 3]. Albeit outbreak of nosocomial infections caused by OXA-48-producing *K. pneumoniae* has been frequently reported in the Mediterranean area and European countries [4–6]. The situation is less common in China, until the nosocomial outbreak of OXA-48-producing *K. pneumoniae* ST147 and ST383 was reported in a Chinese Hospital [7]. Subsequently, clonal dissemination of KPC-2- and OXA-48-coproducing *K. pneumoniae* sequence type 11 and the nosocomial outbreak of OXA-48-producing *K. pneumoniae* caused by clonal expansion of ST11-KL64 sublineages were found in Taiwan [8, 9].

In this study, we provided data on the genomic characterization of an imipenem intermediate *K. pneumoniae* strain isolated from the blood culture of a 56-year-old male patient who suffered from bacteremia and septic shock in May 2017. Antimicrobial susceptibility testing toward imipenem, meropenem, piperacillin, ticarcillin, cefoperazone, cefuroxime, cefazolin, cefoxitin, ampicillin, cefepime, ceftriaxone ceftazidime, amikacin, gentamicin, chloramphenicol, levofloxacin, ciprofloxacin, and trimethoprim/sulfamethoxazole was performed by broth microdilution and that of fosfomycin was determined by the agar dilution method using Mueller Hinton medium supplemented with 25 µg/mL glucose-6-phosphate, and the results were interpreted according to the CLSI 2020 (M100-30ED) guideline [10]. To characterize this strain in detail, whole genome sequencing was further performed using an Illumina MiniSeq plus Pacbio Sequencing. Based on the whole genome sequence, subsequent multi-locus sequence typing (MLST) was determined by uploading the genome to the webtool MLST v2.0 (https://cge.cbs.dtu.dk/services/MLST/), and antimicrobial resistance genes and plasmid replicons were identified by ResFinder 3.2 and PlasmidFinder 2.1 (https://cge.cbs.dtu.dk/services/). The plasmid carrying *bla*_OXA-48_ was detailly characterized by circular plasmid map and comparison of plasmid structures. Additionally, the phylogenomic tree of *bla*_OXA-48_ from global *K. pneumoniae* isolates was further constructed to observe the evolutionary relationship of *bla*_OXA-48_. Briefly, the nucleotide sequences of *bla*_OXA-48_ gene of 576 strains were compared by muscle, and then single nucleotide polymorphism (SNP) sites were used to extract SNPs from multiple alignment species. Finally, the maximum likelihood tree was constructed using RAxML software [11].

Antimicrobial susceptibility testing showed that this strain was intermediate to imipenem (2 μg/mL). However, resistance to meropenem (8 μg/mL) was observed. The isolate was resistant to most tested fluoroquinolones and ß-lactam antimicrobials except cefepime (< 2 μg/mL), ceftriaxone (1 μg/mL), and ceftazidime (4 μg/mL), and susceptible to amikacin (< 4 μg/mL) and gentamicin (< 1 μg/mL) (Table 1). Identification of antimicrobial resistance genes displayed the presence of *bla*_OXA-48_, *fosA5*, and *bla*_SHV-145_. To the best of our knowledge, this is the first time that we reported *K. pneumoniae* ST16 carrying OXA-48 in China, since an NDM-5-producing *K. pneumoniae* isolate also belonging to ST16 has been previously reported [12], thus, we made an in-depth characterization of this strain.

**Table 1:** Antimicrobial susceptibility testing results for the OXA-48-producing *Klebsiella pneumoniae*

Antimicrobials	MIC (μg/mL)^a^	Interpretation^b^
Fosfomycin	> 256	R
Piperacillin	> 128	R
Ticarcillin	> 128	R
Cefoperazone	> 64	R
Cefuroxime	> 32	R
Cefoxitin	> 32	R
Ampicillin	> 32	R
Meropenem	> 16	R
Chloramphenicol	16	R
Levofloxacin	8	R
Cefazolin	> 8	R
Ciprofloxacin	4	R
Ceftazidime	4	S
Imipenem	2	I
Amikacin	< 4	S
Ceftriaxone	1	S
Gentamicin	< 1	S
Cefepime	< 2	S
Trimethoprim/sulfamethoxazole	< 0.5	S

^a^ An antimicrobial susceptibility testing was performed with standard broth microdilution method and interpreted based on the criteria from the Clinical and Laboratory Standards Institute guidelines (M100-30ED-2020)

^b^ *R*, resistant; *S*, susceptible; *I*, intermediate

**Fig. 1:**
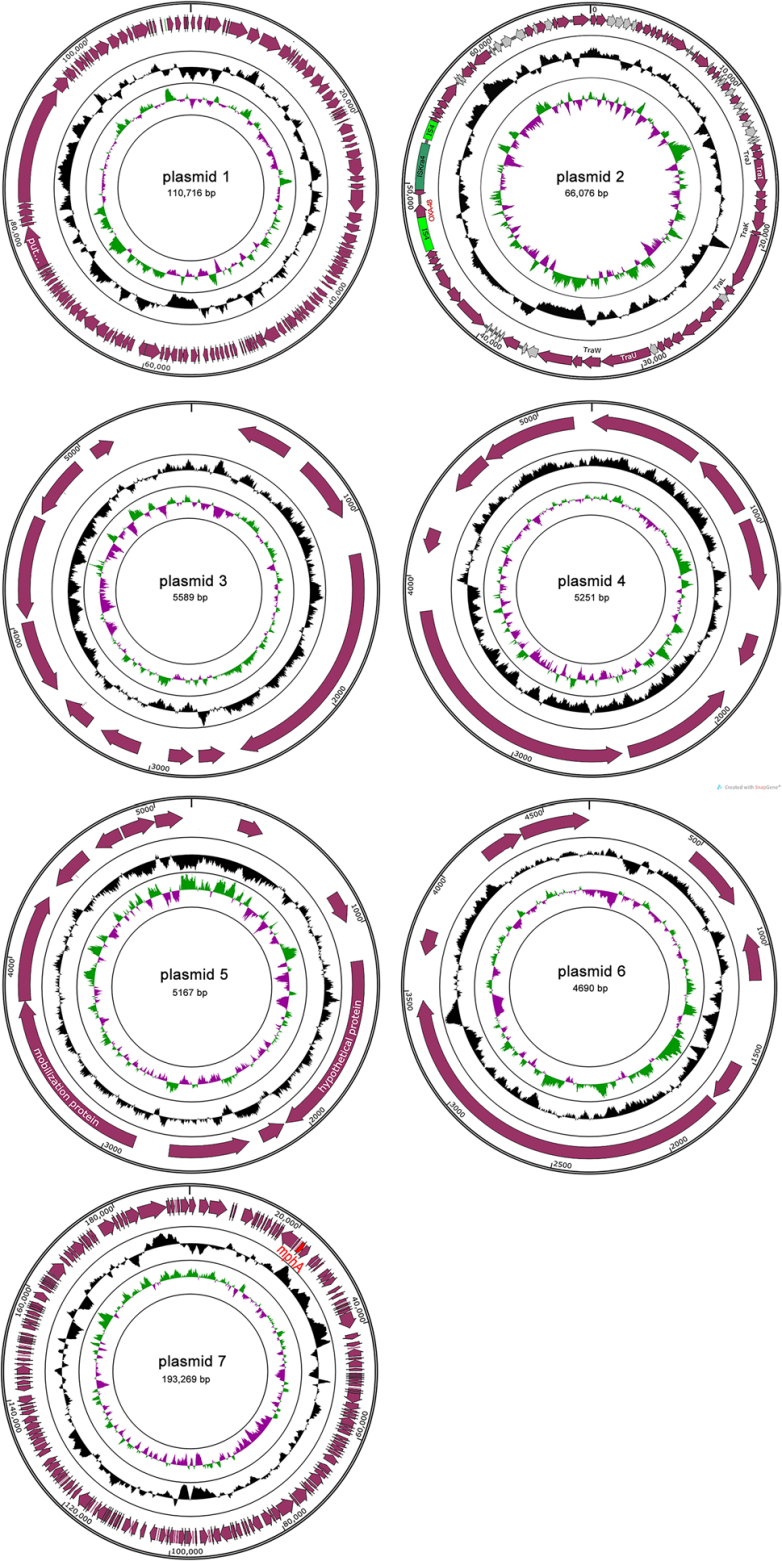
Circular structure of the seven plasmids in the OXA-48-producing *Klebsiella pneumoniae*. The innermost rings represent the G + C skew of the plasmids, the middle rings represent the G + C content, and the outermost rings represent predicted ORFs. Black, content of G + C; claret, open reading frames; light green, insertion sequence

**Fig. 2:**
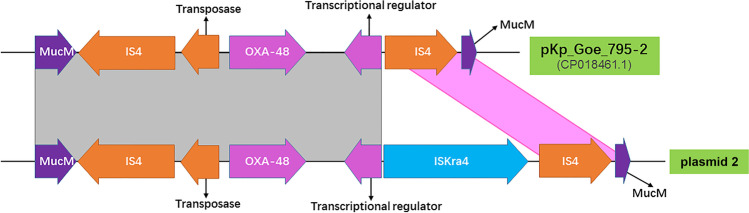
The flanking element analysis of OXA-48 gene between plasmid 2 and its most similar homologue. Colored arrows indicate open reading frames. Orange, blue, and purple arrows represent insertion sequence IS4, ISKra4, and *mucM* genes, respectively. Gray shading indicates homologous regions among different elements

Whole-genome sequencing data by Illumina MiSeq plus Pacbio found that the *K. pneumoniae* isolate contained a 5.68-Mb genome, including a 5.31-Mb chromosome and seven different plasmids (Table 2). Resistant determinants including *fosA5*, *oqxAB*, and *bla*_SHV-145_ were found in chromosome, whereas, *bla*_OXA-48_ was found in a conjugative IncL/M plasmid (Fig. 1). The size of this plasmid is 66,076 bp, with GC content being 55.23% (Fig. 2). The circular structure of plasmid showed that this plasmid is likely to be a conjugative mobile plasmid because of the frequent binding mobile element *tra*-associated operons region within this plasmid [13] (Fig. 1). As known, IncL/M plasmid has been reported to be the vector for *bla*_OXA-48_ [14]. Analysis of flanking elements of *bla*_OXA-48_ gene displayed mobile elements including insertion sequence 4 (IS4), transposase, and IS_kra_4. We found that IS4 was distributed among both of the downstream and upstream of *bla*_OXA-48_ gene (Fig. 2). So far, IS4 family element has been reported to be involved in mobilization and expression of ß-lactam resistance genes including *bla*_VEB-1_ and *bla*_OXA-48_ [15]. Transposase has been found to mediate chromosomal integration of exogenous genes in *Acidithiobacillus ferrooxidans* [16]. Altogether, our study suggested that the *bla*_OXA-48_ gene might be rapidly spread by a broad host-range conjugative plasmid.

**Table 2:** Genomic features of the OXA-48-producing *Klebsiella pneumoniae*

Structure	Length (bp)	GC (%)	Antimicrobial resistance genes	Replicon type	Accession no
Chromosome	5,314,991	57.49	*oqxAB*, *fosA5*, *bla*SHV-145		CP058581
Plasmid 1	110,716	48.84	None	Unknown	CP058582
Plasmid 2	66,076	51.23	OXA-48	IncL/M	CP058583
Plasmid 3	5,589	51.22	None	Unknown	CP058584
Plasmid 4	5,251	49.24	None	IncCoI440II	CP058585
Plasmid 5	5,167	47.57	None	Unknown	CP058586
Plasmid 6	4,693	43.91	None	IncCoI440I	CP058587
Plasmid 7	193,269	52.50	Mph(A)	IncFIB(K) IncFII(K)	CP058588

Noteworthily, this is the first time that we identified SHV-145 in a clinical *K. pneumoniae* isolate in China. SHV-145 is an extended-spectrum beta-lactamase that has been previously found in a *K. pneumonia* clinical isolate recovered from a hospitalized patient in Portugal (unpublished data: accession number AFN88952.1). To date, SHV-145 has been predicted by the protein homolog model (AMR detection model) as a beta-lactamase which could lead to the inactivation of penam, cephalosporin and carbapenem (https://card.mcmaster.ca/ontology/37565#resistomes-table). Furthermore, SHV-ESBLs are usually encoded by self-transmissible plasmids. However, in our study, *bla*_SHV-145_ was identified in Chromosome, which may be mobilized by mobile elements.

**Fig. 3:**
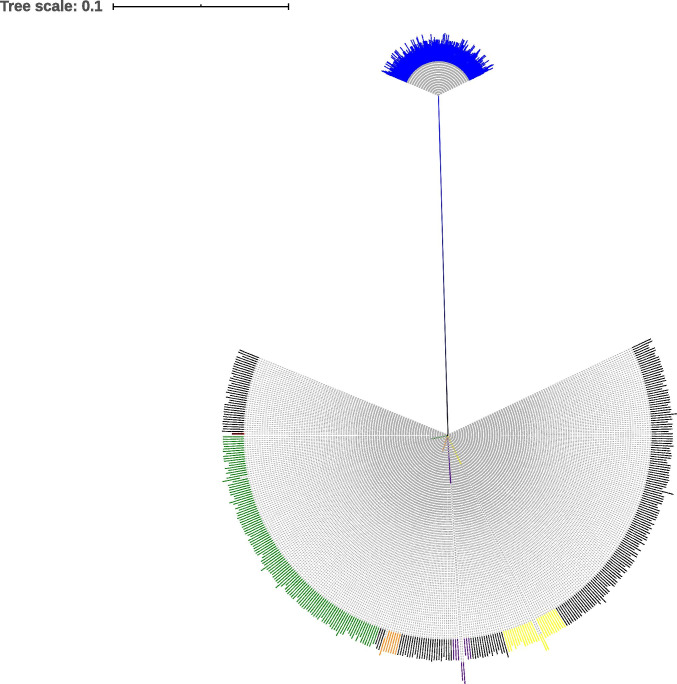
The *bla*_OXA-48_ phylogenetic tree based on the global 576 OXA-48-producing *Klebsiella pneumoniae* isolates. The nucleotide sequences of *bla*OXA-48 gene of 576 strains were compared by muscle, and then single nucleotide polymorphism (SNP) sites were used to extract SNPs from multiple alignment species. The maximum likelihood tree was constructed using RAxML. Red, the OXA-48-producing *K. pneumoniae* isolate in our study

The phylogenetic tree based on the *bla*_OXA-48_ gene from global 576 K*. pneumoniae* isolates displayed 2 clades (Fig. 3). The simple evolutionary relationship suggests that this gene is relatively conservative. Concurrently, this may also indicate that *bla*_OXA-48_ has a strong transmission ability among *K. pneumoniae* isolates.

To the best of our knowledge, this is the first report on the co-occurrence of *fosA5*, *bla*_SHV-145_ and *bla*_OXA-48_ among the *K. pneumoniae* ST16 in China and for the first time, we prescribed the *bla*_OXA-48_ evolutionary phylogenomic of global-producing *K. pneumoniae.*
